# Preparation of Stacked Polymyxin B-Functionalized Cryogels for Efficient Endotoxin Removal from Complex Biological Systems

**DOI:** 10.3390/gels12060470

**Published:** 2026-05-28

**Authors:** Peiji Liu, Yinfeng Wang, Hong Lin, Jingxue Wang

**Affiliations:** College of Food Science and Engineering, Ocean University of China, Huangdao Campus, Qingdao 266400, China; liupeiji@stu.ouc.edu.cn (P.L.); yif_wang1@163.com (Y.W.); linhong@ouc.edu.cn (H.L.)

**Keywords:** cryogel, cryopolymerization, endotoxin, polymyxin B, affinity adsorption

## Abstract

Development of efficient and robust endotoxin removal approaches is vital for safeguarding the safety and functional performance of bacteriophage formulations and recombinant protein bioproducts. Here, a stacked gel-casting strategy combined with carboxymethyl chitosan (CMCS) reinforcement was employed to construct a mechanically robust PMB-functionalized cryogel. The synergistic effects of the stacked architecture and CMCS reinforcement significantly enhanced pore-wall stability and functional site accessibility, resulting in a high endotoxin adsorption capacity (1.88 × 10^6^ EU/g) and excellent reusability (>90.30% removal efficiency after six cycles). The cryogel also demonstrated effective endotoxin removal in complex biological samples, achieving >99.00% removal in bacteriophage preparations with improved phage recovery and >99.99% clearance in recombinant protein solutions. These results highlight a promising strategy for endotoxin control in phage-based applications and biopharmaceutical purification.

## 1. Introduction

In the development and production of bioproducts, including recombinant proteins, antibody formulations, and rapidly emerging bacteriophage preparations, product safety and purity remain critical quality attributes [1,2,3]. In applications such as food safety detection, aquaculture disease control, and cell or animal model studies, even trace levels of impurities may compromise experimental accuracy or introduce potential safety risks [4,5]. For instance, in aquaculture, bacteriophages are increasingly recognized as natural alternatives to antibiotics; however, large-scale production often results in the release of substantial amounts of endotoxins due to bacterial lysis, which may accumulate in the final products, induce stress responses in aquatic organisms, and even pose risks to food safety through the food chain [6,7]. Similarly, in enzyme-linked immunosorbent assay (ELISA)-based analyses, residual endotoxins in recombinant antibodies can elevate background signals and increase false-positive rates, thereby significantly undermining assay reliability [3,8]. These examples highlight that endotoxin contamination not only affects experimental robustness but is also directly linked to the safety evaluation and industrial application of bioproducts.

Despite continuous advances in fermentation engineering and downstream purification technologies, endotoxin contamination remains difficult to eliminate, particularly in processes involving Gram-negative bacterial expression systems [6,9]. Lipopolysaccharides (LPSs), originating from the outer membrane of Gram-negative bacteria, are readily released during bacteriophage amplification, cell disruption, and recombinant protein expression and purification [10,11]. The lipid A moiety confers exceptional chemical stability to LPS, rendering it resistant to conventional inactivation methods such as heat treatment, pH adjustment, or chemical denaturation [12]. Residual endotoxins in bioproducts can trigger strong immune responses in animal studies, interfere with analytical systems, disrupt enzymatic kinetics, and even induce chronic inflammation and tissue damage in aquaculture applications [9]. Therefore, the efficient and reliable removal of endotoxins from complex systems remains a critical bottleneck restricting the broader application of bacteriophage products, recombinant proteins, and antibody-based formulations.

Stringent regulatory standards have been established worldwide to control endotoxin levels in food and pharmaceutical products [13]. For example, the U.S. Food and Drug Administration (FDA) and the European Pharmacopoeia (EP) specify endotoxin limits of 0.25 and 0.5 EU/mL, respectively, for water for injection, while injectable drugs for human use must comply with stricter thresholds (USP ≤ 5 EU/kg; EP ≤ 0.2 EU/kg) [14,15,16]. In addition, endotoxin levels in recombinant proteins used for in vitro cell culture are typically required to be below 1 EU/μg, and even lower thresholds (<0.1 EU/μg) are recommended for in vivo studies [17,18]. Although specific regulatory limits for bacteriophage preparations have not yet been formally established in some regions, expert consensus recommends that endotoxin levels should not exceed 250 EU/10^7^ PFU [19]. These stringent requirements underscore the importance of effective endotoxin control to ensure both safety and efficacy.

However, the removal of endotoxins from real-world samples remains highly challenging due to the complexity of biological matrices, which typically contain proteins, nucleic acids, polysaccharides, lipids, and other components [5,20,21]. For example, high concentrations of DNA and cell debris in phage lysates may compete with endotoxins for binding sites on adsorbent materials [22]; hydrophobic domains or charged regions in protein solutions may shield effective adsorption sites [23]; and lipid nanoparticles or virus-like particles present in vaccine systems can interact non-specifically with LPS, further complicating separation processes [12]. These factors significantly reduce the selectivity and efficiency of conventional endotoxin removal strategies in complex environments, highlighting the urgent need for advanced materials capable of maintaining high performance under such conditions.

To address these challenges, various endotoxin removal strategies have been developed, including anion-exchange chromatography, membrane filtration, two-phase extraction, and affinity adsorption [24,25,26,27]. Among them, affinity materials functionalized with polymyxin B have attracted considerable attention due to their high specificity toward LPS [28,29,30]. Cryogels are a class of macroporous polymeric materials characterized by interconnected pore structures, which enable low flow resistance, high permeability, and efficient mass transfer [31,32]. Previous studies have reported PMB-functionalized cryogels, such as CG (HEMA-co-AM)@ECH@PMB, which exhibit promising endotoxin adsorption performance in model protein systems [23]. The macroporous structure of cryogels facilitates rapid mass transfer and provides accessible channels for LPS binding, while PMB interacts with LPS through combined electrostatic and hydrophobic interactions [32,33]. However, further investigations have revealed that cryogels prepared using conventional monolithic column formats often suffer from structural heterogeneity, including uneven pore distribution, pore wall collapse, and structural fatigue, which ultimately compromise adsorption efficiency and reusability during repeated adsorption–desorption cycles [23].

To overcome these limitations, this study introduces a dual optimization strategy involving both fabrication methodology and material composition. On one hand, a stacked cryogel fabrication approach was developed using pre-cooled multi-well plates, which significantly enhances heat and mass transfer during cryopolymerization, resulting in a more uniform freeze-induced phase separation process and improved structural homogeneity. On the other hand, carboxymethyl chitosan, a hydrophilic and biocompatible polysaccharide with abundant functional groups, was incorporated to reinforce pore wall thickness and enhance overall structural stability [34]. Based on these improvements, a novel PMB-functionalized composite cryogel with enhanced mechanical strength, structural integrity, and suitability for complex system processing was successfully constructed.

In this work, the prepared cryogel was systematically evaluated in terms of structural characteristics, adsorption performance, reusability, and applicability in complex biological matrices. Furthermore, the synergistic contributions of the stacked architecture, CMCS reinforcement, and PMB-specific affinity interactions were elucidated. This study provides an effective and practically applicable strategy for endotoxin removal from complex biological systems, thereby supporting the safe production and broader application of bioproducts, including bacteriophage preparations, recombinant proteins, and vaccines.

## 2. Results and Discussion

### 2.1. Micro-CT Characterization of CG (HEMA-co-AM)@ECH@PMB

**Figure 1:**
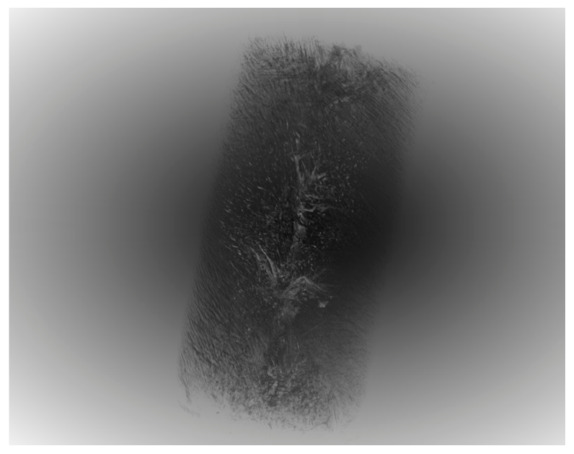
3D image of CG (HEMA-co-AM)@ECH@PMB.

Micro-computed tomography (micro-CT) provides high-resolution three-dimensional imaging, enabling direct visualization of the internal microstructure of porous materials, including pore size, morphology, and spatial distribution [35]. As shown in Figure 1, micro-CT analysis revealed that although CG (HEMA-co-AM)@ECH@PMB has been demonstrated to exhibit effective endotoxin removal in previous studies, structural heterogeneity was still present within the gel matrix after fabrication, freeze-drying, activation, ligand coupling, and repeated adsorption–desorption cycles. This structural deterioration resulted in an uneven pore distribution, particularly characterized by localized densification in the central region of the cryogel.

Such structural heterogeneity may originate from two primary factors. First, intrinsic defects introduced during the initial gel fabrication process may compromise the uniformity of the polymer network, thereby negatively affecting subsequent epoxy functionalization and ligand immobilization efficiency and ultimately limiting endotoxin adsorption capacity. Second, repeated perfusion cycles may induce progressive mechanical fatigue and localized structural deformation within the cryogel matrix, leading to disruption of the pore architecture and consequently reducing both reusability and overall adsorption performance [23]. Therefore, improving the structural integrity of cryogels through rational optimization of both compositional design and architectural configuration is essential for enhancing mechanical stability and sustaining high endotoxin removal efficiency.

### 2.2. Preparation and Structural Property Optimization Analysis of Stacked Cryogel CG (HEMA-co-AM (CMCS))

Due to the relatively large volume and thickness of the monolithic columnar cryogel, heat and mass transfer within the matrix during the low-temperature polymerization stage are inherently slow and spatially heterogeneous. This limitation can impede the complete progression of the crosslinking polymerization reaction, resulting in a relatively loose three-dimensional network skeleton with insufficient overall mechanical strength. Under subsequent fluid perfusion conditions, such structural deficiencies may readily lead to skeletal deformation and instability of the pore architecture.

**Figure 2:**
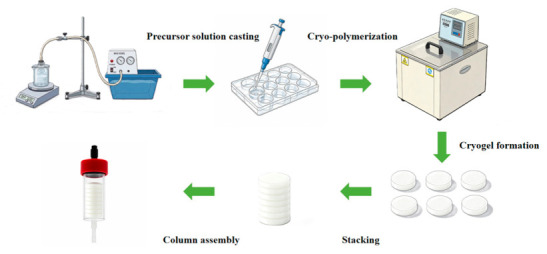
Preparation flowchart of CG (HEMA-co-AM (CMCS))@ECH@PMB.

To address the aforementioned drawbacks, this study optimized the preparation protocol by implementing a stacking-based cryogel fabrication process. The effects of this stacking method on improving the homogeneity and structural stability of the cryogels were systematically investigated; the detailed fabrication process and cryogel morphology are illustrated in Figure 2.

Specifically, a fixed volume of the cryogel precursor solution was added dropwise into pre-cooled 12-well plates. After sealing, the plates were placed in a low-temperature thermostatic bath to undergo cryopolymerization, yielding disc-shaped cryogels with uniform dimensions and morphology. Following epoxy functionalization and ligand coupling, multiple cryogel discs were sequentially stacked and packed into an empty chromatography column. Leveraging the lateral confinement provided by the column walls, a quasi-integrated stacked column bed was constructed.

**Figure 3:**
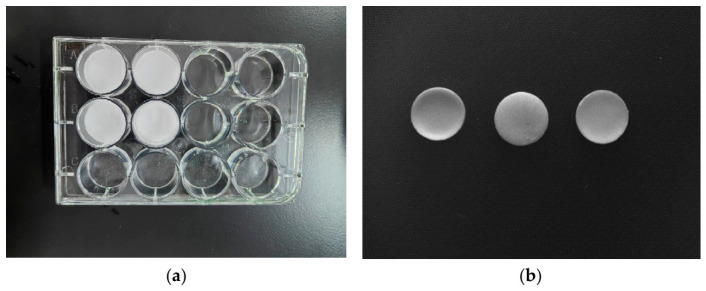
(**a**) Macroscopic view of a cryogel sheet obtained after cryo-polymerization; (**b**) macroscopic view of a cryogel sheet after freeze-drying.

As shown in Figure 3, the stacked cryogels prepared via this method exhibited a plump appearance and uniform structure, with no significant shrinkage observed in the center after lyophilization. Regarding the underlying mechanism, utilizing pre-cooled 12-well plates as reaction vessels effectively increases the specific surface area for heat and mass transfer within the reaction mixture. This significantly enhances the thermal and mass transport efficiency, ensuring that the copolymerization proceeds synchronously and uniformly throughout the cryogel stack [36]. This approach not only fundamentally eliminates incomplete cross-linking but also effectively reduces the formation probability of oversized pores or internal voids, ultimately resulting in high-quality cryogels with a more homogeneous and fine-textured pore structure [37].

Compared with the conventional monolithic columnar structure, the stacked CG (HEMA-co-AM (CMCS)) cryogels prepared in this study possess a more uniform and well-ordered porous architecture, which can provide a larger solid–liquid interfacial area for subsequent epoxy functionalization and ligand coupling processes [38]. This structural advantage is expected to further enhance the efficiency of epoxy functionalization, ligand immobilization, and target molecule adsorption, thereby laying a solid foundation for improving the reusability and overall application performance of the cryogels [39]. The practical resistance of the stacked cryogel column bed to structural collapse, along with the long-term stability of its pore architecture and its reusability, will be systematically evaluated and further validated through subsequent perfusion adsorption and cyclic reuse experiments.

**Figure 4:**
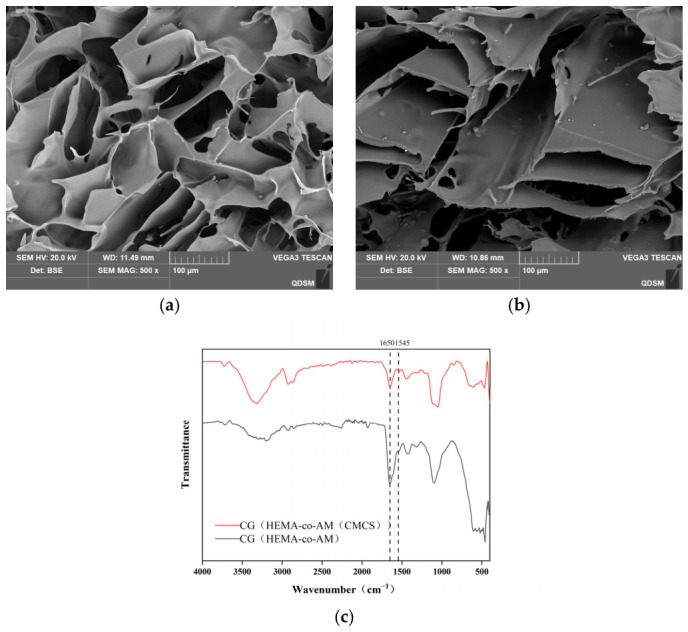
(**a**) Scanning electron microscope (SEM) results of the freeze-dried CG (HEMA-co-AM) sample; (**b**) SEM results of the freeze-dried CG (HEMA-co-AM (CMCS)); (**c**) infrared spectrogram of CG (HEMA-co-AM (CMCS)).

SEM images of freeze-dried samples (Figure 4a,b) show that, compared with CG (HEMA-co-AM), the introduction of carboxymethyl chitosan into CG (HEMA-co-AM (CMCS)) results in a noticeable thickening of the pore walls in the dry state, indicating effective regulation of the microstructure. Such thicker and more loosely structured pore walls suggest enhanced mechanical stability and structural robustness, thereby providing a potential structural basis for improved durability in subsequent application tests [40,41]. While SEM imaging of freeze-dried specimens is a widely adopted characterization approach in cryogel research, it should be noted that these images primarily serve for relative morphological comparison between different materials in the dry state, providing supporting evidence for the pore-wall reinforcing effect of CMCS incorporation rather than serving as an absolute representation of the internal structure of the gel in its swollen state.

Fourier transform infrared (FT-IR) spectra (Figure 4c) indicate that CG (HEMA-co-AM (CMCS)) retains the characteristic absorption peaks of CG (HEMA-co-AM), including 1080 cm^−1^ (C–O–C stretching vibration) and 1650 cm^−1^ (amide I band, C=O stretching). The absorption peak at 1545 cm^−1^ corresponds to the amide II band, associated with the coupling of N–H bending and C–N stretching vibrations [42,43]. Compared with the unmodified gel, the intensity of this peak is significantly enhanced after CMCS incorporation. Additionally, the O–H stretching vibration near 3434 cm^−1^ is also strengthened, suggesting enhanced hydrogen bonding interactions within the system, likely due to interactions between CMCS and polyacrylamide chains [44]. These results confirm the successful incorporation of CMCS into the AM–HEMA copolymer network and demonstrate that enhanced intermolecular interactions contribute to improved structural stability. Therefore, the introduction of CMCS represents an effective strategy for structural regulation in acrylamide-based cryogel systems, enabling the fabrication of functional materials with both large pore structures and improved mechanical strength.

Under the optimized conditions for epoxy functionalization and coupling established in previous studies, stacked CG (HEMA-co-AM (CMCS)) was successfully functionalized to obtain CG (HEMA-co-AM (CMCS))@ECH@PMB. As summarized in Table 1, the epoxy group density of the stacked cryogel reached 41.17 μmol/g, representing an approximately 19.32% increase compared with the monolithic CG (HEMA-co-AM). This result highlights the advantage of the stacked structure in increasing the density of active sites. Furthermore, the PMB ligand density of CG (HEMA-co-AM (CMCS))@ECH@PMB was also significantly higher than that of the control group, with an increase of approximately 22.30%. This enhancement is mainly attributed to the larger contact area provided by the stacked structure, which facilitates more efficient interaction between the gel surface and activation/coupling reagents, thereby improving the overall reaction efficiency.

### 2.3. Functionalization and Characterization of Stacked CG (HEMA-co-AM (CMCS))

**Figure 5:**
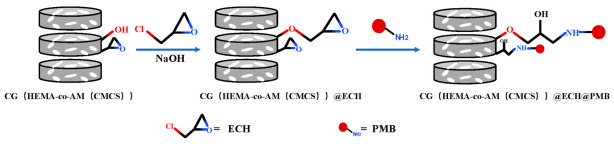
Schematic diagram of the preparation of CG (HEMA-co-AM (CMCS))@ECH@PMB.

Following the efficient fabrication of the stacked CG (HEMA-co-AM (CMCS)) cryogels, functional modification was performed to impart the material with specific endotoxin adsorption capacity. Leveraging the inherent functional group characteristics of the cryogel matrix, the functionalization was achieved through a two-step sequential chemical process involving epoxy functionalization and ligand coupling. The detailed chemical reaction pathways are illustrated in Figure 5.

In the stacked cryogel CG (HEMA-co-AM (CMCS)) prepared in this study, allyl glycidyl ether (AGE) was introduced during the polymerization process to graft initial epoxy groups into the polymer backbone. In addition, the cryogel inherently contains abundant hydroxyl groups [34]. Preliminary experimental results demonstrated that although AGE can introduce a certain amount of epoxy functionalities into the copolymer system, the overall loading remains limited [23]. Simply increasing the AGE content to enhance the surface epoxy density and subsequent ligand coupling efficiency leads to pronounced adverse effects. Specifically, with increasing AGE feed ratio, the mechanical properties—including toughness, elasticity, and structural integrity—continuously deteriorate, when the AGE content exceeds 20.00% of the total monomer mass, the three-dimensional network becomes unstable, resulting in pore structure collapse and even failure of gel formation [23,45,46]. Therefore, the optimized ratio between AGE and monomer components was determined based on literature reports and preliminary optimization experiments in this study.

To further address the insufficient density of active epoxy groups, epichlorohydrin (ECH) was employed as an activating agent to modify the hydroxyl-rich cryogel framework. Under alkaline conditions, the hydroxyl groups on the cryogel backbone are deprotonated to form highly reactive alkoxide intermediates, which subsequently undergo nucleophilic substitution with epichlorohydrin [47]. Through covalent grafting, a large number of epoxy functionalities are introduced onto the polymer network, yielding epoxy- functionalized cryogels denoted as CG (HEMA-co-AM (CMCS))@ECH. This secondary activation strategy significantly increases the density of reactive sites on the cryogel surface without compromising its original physical structure or mechanical performance, thereby providing sufficient binding sites for subsequent ligand immobilization and establishing a robust foundation for functional modification [48].

On this basis, PMB was further covalently immobilized onto the activated cryogel. The abundant epoxy groups on the surface of CG (HEMA-co-AM (CMCS))@ECH exhibit high reactivity toward nucleophilic attack by amino groups in the molecular structure of PMB, enabling efficient covalent coupling and stable immobilization of PMB within the three-dimensional cryogel matrix [23]. The resulting functionalized cryogel was designated as CG (HEMA-co-AM (CMCS))@ECH@PMB.

The endotoxin adsorption capability of the functionalized cryogel is primarily attributed to the covalently immobilized PMB. The lipid A moiety, which represents the toxic core structure of endotoxin, exhibits strong and specific affinity toward polymyxin B [23,49]. Based on this highly specific molecular interaction, the modified cryogel can selectively recognize target endotoxins and achieve efficient and stable adsorption, providing a reliable material platform for effective endotoxin removal.

### 2.4. Evaluation of Endotoxin Adsorption in Tris-HCl Buffer and Model Protein Solutions

**Figure 6:**
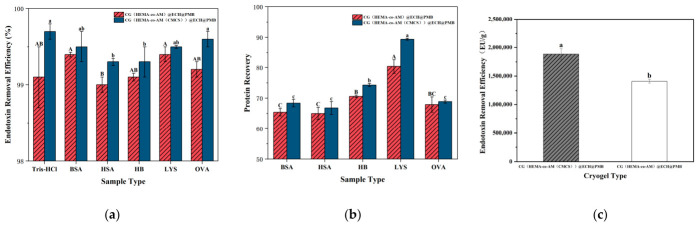
(**a**) LPS adsorption capacity of CG (HEMA-co-AM (CMCS))@ECH@PMB in different types of samples; (**b**) sample elution capacity of CG (HEMA-co-AM (CMCS))@ECH@PMB in different types of samples; (**c**) maximum adsorption capacity of CG (HEMA-co-AM (CMCS))@ECH@PMB. Different lowercase letters indicate significant differences within the same group (*p* < 0.05); different uppercase letters indicate significant differences between groups (*p* < 0.05).

To evaluate the endotoxin adsorption performance of stacked CG (HEMA-co-AM (CMCS))@ECH@PMB, perfusion experiments were conducted using Tris-HCl buffer and five model protein solutions (Figure 6a). The results indicate that both monolithic CG (HEMA-co-AM)@ECH@PMB and stacked CG (HEMA-co-AM (CMCS))@ECH@PMB exhibit similar adsorption trends; however, the stacked cryogel demonstrates superior endotoxin removal efficiency. In Tris-HCl buffer, the endotoxin removal rate of the stacked cryogel reached 99.70%. In all five protein solutions, the adsorption efficiency of the stacked cryogel was consistently higher than that of the monolithic counterpart, highlighting its enhanced capability for endotoxin removal in complex systems.

Further analysis revealed significant differences in structural design and performance between the stacked and monolithic cryogels. The internal diameter of the stacked cryogel increased from 13 mm to 22.45 mm, resulting in a substantially higher maximum flow rate. However, the increased diameter and base area led to a reduced column height at the same mass, thereby shortening the contact time between the sample and the gel, which would theoretically reduce endotoxin adsorption efficiency. Despite this, the stacked cryogel exhibited superior epoxy functionalization and ligand coupling efficiency, enabling the immobilization of a greater amount of polymyxin B per unit mass. As a result, the overall endotoxin adsorption performance was not compromised but instead slightly improved.

In addition, the stacked CG (HEMA-co-AM (CMCS))@ECH@PMB demonstrated improved protein recovery compared with the monolithic CG (HEMA-co-AM)@ECH@PMB (Figure 6b). Among the five protein samples tested, lysozyme (LYS) exhibited the highest recovery, with an elution yield of 89.42% after a single treatment. This improvement can be attributed to the reduced contact time between proteins and the gel matrix in the stacked structure, which minimizes non-specific adsorption and enhances protein recovery.

Regarding adsorption capacity, the stacked CG (HEMA-co-AM (CMCS))@ECH@PMB exhibited outstanding performance owing to its elevated PMB loading. The maximum endotoxin adsorption capacity reached 1,883,466.32 EU/g, representing an approximately 25.22% increase compared with CG (HEMA-co-AM)@ECH@PMB (Figure 6c). This value ranks among the highest reported for endotoxin adsorbents to date, exceeding those reported for PMB-functionalized materials based on other matrices, such as cellulose microspheres or electrospun nanofibers [10,28]. The capacity advantage over adsorbents employing alternative ligands, including polyethyleneimine-grafted microspheres and chitosan-based nanofibrous membranes [50,51], is also substantial. This enhancement is primarily attributed to the synergistic effect of the stacked gel-casting strategy and CMCS reinforcement, which together provide a higher pore-wall surface area for ligand immobilization and consequently superior PMB loading [52]. This result highlights the excellent performance of the stacked CG (HEMA-co-AM (CMCS))@ECH@PMB in endotoxin removal and provides both theoretical and practical support for its application in adsorption-based separation technologies.

**Figure 7:**
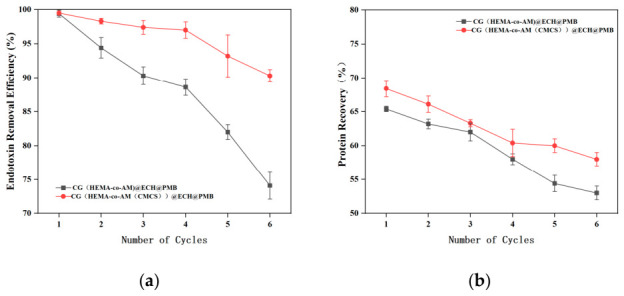
(**a**) The effect of usage times on the LPS adsorption capacity of CG (HEMA-co-AM (CMCS))@ECH@PMB; (**b**) the effect of usage times on the sample elution of CG (HEMA-co-AM (CMCS))@ECH@PMB.

During repeated use, the internal pore walls of the cryogel may be damaged by continuous perfusion, leading to a gradual decline in adsorption performance. As shown in Figure 7, after six consecutive adsorption cycles, the endotoxin removal efficiency of the stacked CG (HEMA-co-AM (CMCS))@ECH@PMB decreased slightly but remained above 90.00%. In contrast, the adsorption efficiency of CG (HEMA-co-AM)@ECH@PMB dropped to 74.12% in the sixth cycle. Moreover, the stacked cryogel maintained stable protein recovery, exhibited no structural collapse, and caused no secondary contamination, demonstrating excellent stability and reliability. In comparison, while several cryogel- and nanoparticle-based adsorbents have shown reusability potential, the demonstrated high retention of removal efficiency beyond 90.00% over six consecutive cycles is noteworthy [53,54]. This robust reusability is ascribed to the structural integrity conferred by CMCS reinforcement, which effectively mitigates pore-wall damage during prolonged operation. Previous studies have demonstrated that CMCS can significantly enhance the mechanical properties of gels by forming interpenetrating networks or providing structural support, thereby improving fatigue resistance and reusability stability [55,56].

The significantly improved durability and reusability of the optimized cryogels can be fundamentally attributed to the enhanced microstructure revealed by SEM analysis. Specifically, the thickened pore walls serve as the primary load-bearing framework, which can more effectively withstand and dissipate the stresses generated during repeated use processes, such as cyclic adsorption–desorption. This structural reinforcement helps to prevent common failure modes associated with thin-walled architectures, including buckling and fracture.

Meanwhile, the relatively enlarged pore size facilitates mass transfer during operation and alleviates pore blockage, thereby further contributing to stable performance under dynamic conditions. Therefore, the observed increase in pore wall thickness and pore size in the SEM images should not be regarded as isolated morphological variations, but rather as a direct microstructural basis underlying the enhanced structural stability and reusability [44]. This robust porous network ultimately ensures sustained high performance during long-term applications. The synergistic effect of these factors enables the cryogel to maintain high endotoxin adsorption efficiency even after multiple reuse cycles, providing a robust and efficient material platform for processing complex biological samples [23,34].

### 2.5. Evaluation of Endotoxin Removal in Practical Samples

**Figure 8:**
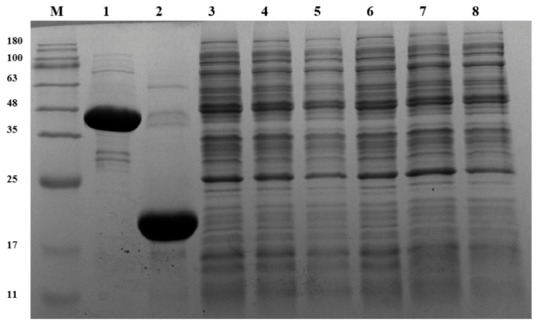
SDS-PAGE of recombinant proteins (1. AK; 2. SCP; 3. G20B (induced at 20 °C); 4. G20B (induced at 30 °C); 5. G20B (induced at 37 °C); 6. G37B (induced at 20 °C); 7. G37B (induced at 30 °C); 8. G37B (induced at 37 °C)).

To assess the endotoxin removal performance of stacked CG (HEMA-co-AM (CMCS))@ECH@PMB in high-concentration recombinant protein samples, four proteins with well-established laboratory expression systems and high yields were selected and successfully produced using an Escherichia coli expression system [57,58]. As shown in Figure 8, protein AK exhibited a molecular weight of approximately 42 kDa with a clear electrophoretic band, indicating high purity and structural integrity, whereas protein SCP showed a lower molecular weight of approximately 24 kDa.

To further investigate adsorption behavior in complex systems, G20B and G37B proteins expressed at different induction temperatures (20 °C, 30 °C, and 37 °C) were used without purification. These proteins exhibited relatively high concentrations, with improved expression observed under low-temperature induction (20 °C) (Table 2). The prepared recombinant protein samples were subsequently subjected to perfusion treatment for endotoxin removal.

In practical applications, recombinant protein samples typically contain complex components, including target proteins, endotoxins, and various impurities, which often result in reduced treatment efficiency compared with simple protein solutions. As shown in Table 3, after three consecutive treatment cycles, stacked CG (HEMA-co-AM (CMCS))@ECH@PMB successfully reduced the endotoxin levels of eight protein samples to below 2.5 EU/mL, achieving an endotoxin removal efficiency exceeding 99.99%. In contrast, recent studies have reported that conventional affinity adsorbents or polysaccharide-based materials typically exhibit endotoxin removal efficiencies ranging from 70.00% to 90.00% in complex biological systems due to diffusion limitations and non-specific competitive interactions [1,59]. Therefore, the composite cryogel developed in this study demonstrates a significant advantage in endotoxin removal from complex recombinant protein samples.

**Figure 9:**
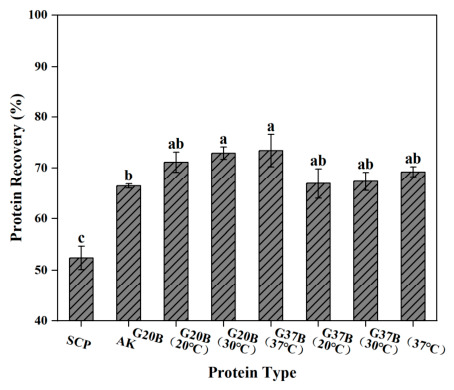
Measurement of the effect of CG (HEMA-co-AM (CMCS))@ECH@PMB treatment on recovery in recombinant protein samples. Different letters (a, b, c) on the columns indicated significant difference between each other at *p* < 0.01 level.

Protein recovery is influenced by multiple factors, including the intrinsic physicochemical properties of the protein as well as the complexity of the solution matrix. As shown in Figure 9, except for the SCP group, the recovery yields of the remaining seven proteins were all higher than 66.44%. In contrast, previous studies have demonstrated that conventional endotoxin removal methods, such as surface adsorption and Triton X-114 phase separation, are often associated with substantial protein loss in complex systems. Their recovery yields are typically reduced due to non-specific adsorption and partitioning effects at phase interfaces [26,60]. Therefore, the cryogel developed in this study not only achieves highly efficient endotoxin removal but also better preserves protein activity and recovery, demonstrating a clear advantage over conventional methods.

For the same type of recombinant proteins (G20B and G37B), the protein recovery showed a decreasing trend with increasing protein concentration. This phenomenon can be attributed to the fact that higher protein concentrations are more likely to induce non-specific adsorption onto the cryogel matrix, slight pore blockage, and the formation of protein–endotoxin complexes. Notably, SCP protein, with an isoelectric point of approximately 4.67 and relatively strong hydrophobicity [58], exhibited more pronounced non-specific interactions with PMB. As a result, despite its relatively low concentration and simpler system composition, SCP showed a comparatively low recovery yield (only 52.42%).

After three consecutive endotoxin removal cycles for eight recombinant protein samples, the endotoxin levels of all samples were reduced to below 1 EU/mg, fully meeting the safety requirements for in vitro cell-based assays [19]. These results demonstrate that stacked CG (HEMA-co-AM (CMCS))@ECH@PMB exhibits excellent performance in processing complex recombinant protein samples.

**Figure 10:**
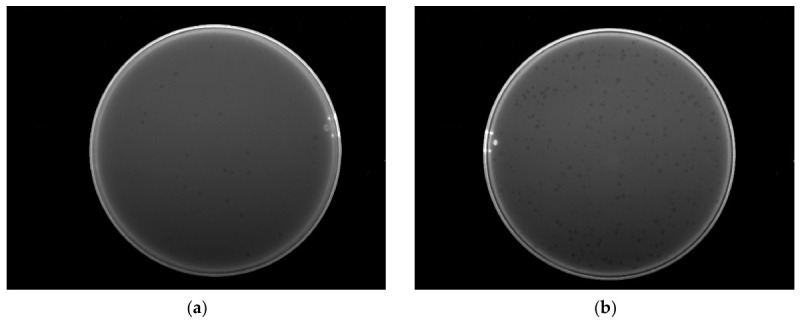
(**a**) Titer determination of HD1 phage before adsorption test; (**b**) titer determination of HD1 phage after adsorption test.

To further evaluate the endotoxin removal capability of stacked CG (HEMA-co-AM (CMCS))@ECH@PMB in bacteriophage systems, nine bacteriophage preparations (covering three phage types) were investigated. Figure 10 illustrates the changes in the titer of HD1 bacteriophage before and after treatment. As shown in Table 4, after a single treatment cycle, the endotoxin removal efficiency for all bacteriophage samples exceeded 99.00%.

In comparison, previous studies have reported that conventional ultrafiltration and gel filtration methods typically achieve endotoxin removal efficiencies of over 90.00% in complex biological systems, with inherent limitations associated with membrane retention effects and separation resolution [27,57]. Therefore, the stacked composite cryogel developed in this study demonstrates superior endotoxin removal efficiency in bacteriophage preparations while effectively preserving phage bioactivity.

**Figure 11:**
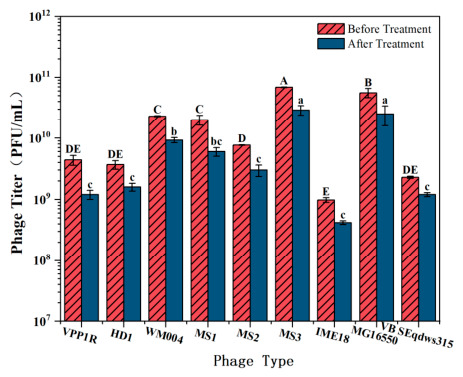
The effect of CG (HEMA-co-AM (CMCS))@ECH@PMB treatment on the titer of phage preparation. Different letters (a, b, c) and (A, B, C, D, E) on the columns indicated significant difference between each other at *p* < 0.05 level.

Following a single endotoxin adsorption cycle using CG (HEMA-co-AM (CMCS))@ECH@PMB, the changes in bacteriophage titers are presented in Figure 11. A decrease in recovery was observed for all bacteriophage samples after treatment. Specifically, the recovery yields of VPP1R, HD1, WM004, MS1, MS2, MS3, IME18, MG16550, and VB SEqdws 315 were 27.27%, 43.24%, 40.43%, 30.00%, 38.96%, 42.03%, 42.86%, 44.64%, and 52.17%, respectively. Although these values are lower than those reported for ultrafiltration methods [22], the present cryogel system exhibited superior performance in terms of endotoxin removal efficiency. Compared with conventional gel filtration and activated carbon adsorption methods, the proposed approach demonstrates an overall performance advantage, particularly in endotoxin clearance.

The reduction in bacteriophage recovery can be attributed to several factors. On one hand, non-specific adsorption of bacteriophages onto the cryogel matrix may occur, likely driven by electrostatic and hydrophobic interactions. On the other hand, during preparation and perfusion, a fraction of bacteriophages may associate with endotoxins to form complexes. Due to the high specificity of CG (HEMA-co-AM (CMCS))@ECH@PMB toward endotoxins, these endotoxin-bound bacteriophages are co-adsorbed by the gel, resulting in reduced phage recovery in the effluent. This mechanism reasonably explains the relatively low recovery observed after a single treatment cycle [59].

Quantitative analysis further revealed that, after one treatment cycle, the endotoxin-to-phage ratios for VPP1R, HD1, WM004, MS1, MS2, MS3, IME18, MG16550, and VB SEqdws 315 were 2.02 EU/10^7^ PFU, 7.75 EU/10^8^ PFU, 2.47 EU/10^9^ PFU, 2.97 EU/10^8^ PFU, 2.90 EU/10^8^ PFU, 3.40 EU/10^10^ PFU, 5.43 EU/10^7^ PFU, 1.28 EU/10^9^ PFU, and 6.58 EU/10^8^ PFU, respectively. All values were significantly below the recommended endotoxin limit for bacteriophage preparations (≤250 EU/10^7^ PFU) as proposed in the Expert Consensus on Phage Therapy in China [19], indicating a high level of safety for the treated products.

Overall, these results demonstrate that stacked CG (HEMA-co-AM (CMCS))@ECH@PMB exhibits excellent performance in endotoxin removal from bacteriophage preparations and holds significant potential for practical applications.

### 2.6. Effect of Storage Conditions on Cryogel Performance

**Figure 12:**
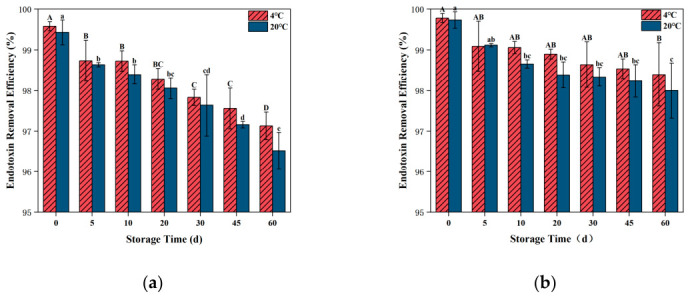
(**a**) The impact of lyophilization and plastic packaging environment on the LPS adsorption capacity of CG (HEMA-co-AM (CMCS))@ECH@PMB (**b**) The effect of 20.00% ethanol environment on the LPS adsorption capacity of CG (HEMA-co-AM (CMCS))@ECH@PMB. Different letters (a, b, c, d, e) and (A, B, C, D) on the columns indicated significant difference between each other at *p* < 0.05 level.

Appropriate storage conditions are crucial for maintaining the performance of stacked CG (HEMA-co-AM (CMCS))@ECH@PMB. As shown in Figure 12, when the cryogel was stored in 20.00% ethanol solution at 4 °C, its endotoxin adsorption efficiency decreased by only 0.90% over 60 days.

In contrast, long-term storage via freeze-drying and vacuum sealing significantly affected the gel structure, leading to internal dehydration, reduced rehydration capacity, and decreased endotoxin adsorption efficiency (declining to 96.52% after 60 days at 20 °C) [61]. The use of 20.00% ethanol as a storage medium not only helps maintain structural stability but also inhibits the growth of bacteria and fungi, preserves moisture balance, and prevents microbial degradation [62].

Furthermore, ethanol exerts a mild fixation effect during storage, contributing to the stabilization of the gel structure and facilitating subsequent experimental handling [63]. Therefore, storage in 20.00% ethanol at 4 °C is considered an optimal strategy for preserving the performance of stacked CG (HEMA-co-AM (CMCS))@ECH@PMB, effectively maintaining its endotoxin adsorption capacity over at least 60 days.

## 3. Conclusions

In this study, individually fabricated cryogel monoliths were assembled into a stacked architecture, combined with carboxymethyl chitosan incorporation, to engineer a structurally reinforced PMB-functionalized cryogel. This design effectively alleviated internal polymerization stress, enhanced pore-wall robustness, and substantially improved the cryogel’s tolerance to complex biochemical environments. The optimized cryogel exhibited a high maximum endotoxin adsorption capacity of 1,883,466.32 EU/g and maintained over 90.30% of its adsorption efficiency after six regeneration cycles, demonstrating excellent reusability and structural stability.

When applied to practical purification systems, the cryogel achieved >99.00% endotoxin removal in bacteriophage formulations while preserving high phage recovery. Similarly, in recombinant protein purification, a three-cycle treatment resulted in >99.99% endotoxin clearance, meeting stringent endotoxin limits required for cell-based assays. Collectively, the CMCS-enhanced cryogel demonstrates superior affinity performance, mechanical robustness, and environmental resilience, highlighting its strong potential as a high-efficiency endotoxin purification platform for bacteriophage preparations, biopharmaceutical production, and other applications requiring rigorous endotoxin control.

## 4. Materials and Methods

### 4.1. Materials

Acrylamide (AM, 98%, Sigma-Aldrich, St. Louis, MO, USA), N,N′-Methylenebisacrylamide (MBAm, 99%, Sigma-Aldrich, St. Louis, MO, USA), Epichlorohydrin (ECH, 99%, Sinopharm, Beijing, China), N,N,N′,N′-Tetramethylethylenediamine (TEMED, 99%, Sigma-Aldrich, St. Louis, MO, USA), Ammonium persulfate (APS, 98%, Sigma-Aldrich, St. Louis, MO, USA), Allyl glycidyl ether (AGE,97%, Sigma-Aldrich, St. Louis, MO, USA), 2-Hydroxyethyl methacrylate (HEMA,99%, Macklin Biochemical, Shanghai, China), Tris(hydroxymethyl)aminomethane (Tris, 99%, Sigma-Aldrich, St. Louis, MO, USA), Sinopharm, Beijing, China) Dimethyl sulfoxide (DMSO, 99.9%, Sinopharm, Beijing, China), Ammonia solution (NH_3_·H_2_O, 25–28%, Sinopharm, Beijing, China), Acetone (99.5%, Sinopharm, Beijing, China), Glucose (99.5%, Sinopharm, Beijing, China) Polymyxin B (PMB, 95% and 10 000 IU/mg activity, Solarbio, Beijing, China), Phenol (99%, Solarbio, Beijing, China), Polyethylene glycol 6000 (PEG6000, 99%, Solarbio, Beijing, China), DNase I (1000 U/mg, Solarbio, Beijing, China), RNase I (100 U/mg, Solarbio, Beijing, China), Proteinase K (30 U/mg, Tiangen, Beijing, China), Coomassie Brilliant Blue R-250 (Solarbio, Beijing, China), Potassium bromide (KBr, 99%, Macklin Biochemical, Shanghai, China), Human serum albumin (HSA, 96%, Solarbio, Beijing, China), Hemoglobin (Hb, 95% heme content 90%, Solarbio, Beijing, China), Lysozyme (LYS, 90%, Solarbio, Beijing, China), Ovalbumin (OVA, 98%, Solarbio, Beijing, China), and Bovine serum albumin (BSA, 98%, Solarbio, Beijing, China) and Carboxymethyl Chitosan (CMCS, 543.52 g/mol, 90%, Solarbio, Beijing, China) were used as received. Deionized water was employed throughout.

Bacteriophages VPP1R, HD1, WM004, MS1, MS2, MS3, IME18, MG16550, and VB SEqdws 315 were isolated from mixed sewage samples collected from Qingdao Nanshan aquatic market, Qingdao sewage treatment plant, and Lianxin Fishing Port, and preserved at the Food Safety Laboratory, Ocean University of China. The plasmids pET-28a-SUMO and pET-28a-TM were purchased from Novagen (Madison, WI, USA). Escherichia coli BL21 (DE3) was obtained from Vazyme Biotech Co., Ltd., Nanjing, China. Deionized water was used throughout the experiments.

### 4.2. Preparation of Stacked Cryogel CG (HEMA-co-AM) (CMCS)@ECH@PMB

#### 4.2.1. Preparation of Stacked CG (HEMA-co-AM) (CMCS)

The stacked CG (HEMA-co-AM) (CMCS) was prepared based on a previously reported method with slight modifications. Briefly, 127.9 mg of acrylamide (Am), 117.1 μL of 2-hydroxyethyl methacrylate (HEMA), 65.2 mg of N,N′-methylenebisacrylamide (MBAm), and 50 mg of carboxymethyl chitosan (CMCS) (total concentration: 1%) were dissolved in 4.5 mL of deionized water together with 40.8 μL of allyl glycidyl ether (AGE) to form solution A. The solution was thoroughly stirred and degassed in an ice bath to ensure system purity.

Separately, 6 mg of ammonium persulfate (APS) was dissolved in ultrasonically treated deionized water to obtain 0.5 mL of solution B. Subsequently, 7.5 μL of N,N,N′,N′-tetramethylethylenediamine (TEMED) was added to solution A under ice-bath conditions, followed by vacuum stirring at 300 r/min for 15 min to remove dissolved gases. Solution A and solution B were then mixed thoroughly and subjected to vacuum degassing in an ice bath for an additional 1.5 min.

The mixture was quickly transferred into a pre-cooled 12-well plate, sealed with a cover and parafilm, and placed in a thermostatic bath at −13 °C for 24 h to allow for polymerization. After polymerization, the cryogel was thawed at room temperature and repeatedly washed with deionized water to remove unreacted monomers. Finally, the gel was pre-cooled at −80 °C and freeze-dried for subsequent use.

#### 4.2.2. Epoxy Functionalization with Epichlorohydrin

A 0.5 g (dry weight) sample of CG (HEMA-co-AM) (CMCS) cryogel was weighed and thoroughly washed with deionized water to remove surface impurities and loosely bound ions. The cryogel was then transferred into an Erlenmeyer flask and immersed in 30 mL of sodium hydroxide solution (pH 8.0), ensuring complete exposure to an alkaline environment.

Subsequently, 1.2 mL of epichlorohydrin (ECH) was added to the system, along with an appropriate amount of dimethyl sulfoxide (DMSO) to enhance the miscibility between ECH and the cryogel matrix. The resulting epoxy- functionalized cryogel was denoted as CG (HEMA-co-AM) (CMCS)@ECH.

#### 4.2.3. Conjugation of Polymyxin B

A 5 mL aliquot of CG (HEMA-co-AM) (CMCS)@ECH was added to 30 mL of polymyxin B solution with an initial concentration of 20 mg/mL. The pH of the system was adjusted to 9.0, and the reaction was carried out at 37 °C with shaking at 120 rpm for 24 h to complete ligand coupling.

After the coupling reaction, the product was transferred into 30 mL ethanolamine solution (1 mol/L, pH 9.0) and further reacted under the same conditions at room temperature for 4 h to block the remaining unreacted epoxy groups.

Finally, the obtained cryogel was thoroughly washed with deionized water and stored in 20% ethanol solution at 4 °C. The final product was denoted as CG (HEMA-co-AM) (CMCS)@ECH@PMB.

### 4.3. Evaluation of Endotoxin Removal from Model Protein Solutions

#### 4.3.1. Perfusion Adsorption and Elution Procedure

A total of 0.5 g of layered CG (HEMA-co-AM) (CMCS)@ECH@PMB was packed into a Flash chromatography column (inner diameter 22.45 mm, Shanghai Xinhu Experimental Equipment Co., Ltd., Shanghai, China). The gels were stacked layer-by-layer to ensure uniform distribution without air bubbles, followed by thorough equilibration with an adequate volume of PBS buffer (pH 7.4).

The column inlet was connected to a 50 mL sample reservoir, and the outlet was connected to a peristaltic pump with a flow rate set at 6 mL/min. The sample solution was passed through the column at a constant flow rate of 6 mL/min, and the effluent was collected. After the sample was completely loaded, the column was washed with PBS buffer (pH 7.4) at three times the gel volume.

The above perfusion–washing process was repeated three times to complete one full cycle of endotoxin adsorption.

After each adsorption cycle, an elution procedure was performed. First, 2 mol/L NaCl solution was pumped through the column to desorb the bound endotoxins, and the eluate was collected. Subsequently, 0.2 mol/L NaCl solution and 20 mmol/L PBS buffer (pH 7.4) containing 1% sodium deoxycholate were sequentially applied to remove non-specifically adsorbed proteins and bacteriophage impurities. Finally, the column was thoroughly rinsed with pyrogen-free water, and all eluates were collected.

After elution, the column was re-equilibrated with PBS buffer (pH 7.4) to regenerate the gel. Once the buffer had completely drained, the gel was stored in 20% (*v*/*v*) ethanol at 4 °C for subsequent use.

After each cycle, the endotoxin concentration and protein concentration in the effluent were measured.

#### 4.3.2. Experimental Design for Evaluating the Effect of Reuse Cycles

Under otherwise identical conditions, 50 mL of BSA solution (protein concentration: 10 mg/mL; endotoxin concentration: 500 EU/mL) was used to investigate the effect of repeated adsorption cycles on endotoxin removal performance of CG (HEMA-co-AM (CMCS))@ECH@PMB.

The perfusion flow rate was maintained at 6 mL/min, and every three consecutive perfusion steps constituted one complete adsorption cycle. After each cycle, the residual sample eluted using 0.2 mol/L NaCl solution was reintroduced into the collected effluent to maintain mass balance. A total of six consecutive adsorption cycles were performed. After each cycle, the endotoxin concentration and protein concentration in the effluent were determined to evaluate the variation in adsorption performance with increasing reuse cycles.

### 4.4. Preparation of Bacteriophage Preparations

The propagation, purification, and titer determination of Salmonella phages (MS1, MS2, MS3), Escherichia coli phages (IME18, MG16550, VB SEqdws315), and Vibrio parahaemolyticus phages (VPP1R, HD1, WM004) were performed according to the method reported by Ning [64].

### 4.5. Preparation of Recombinant Proteins

The expression of recombinant nanobody proteins was performed based on the work of Liu with minor modifications [57]. The expression of AK and SCP proteins was adapted from the work of Zhu [58].

### 4.6. Micro-CT Characterization of Cryogel Structure

The CG (HEMA-co-AM)@ECH@PMB cryogel samples, after three rounds of perfusion treatment, were subjected to high-resolution X-ray micro-computed tomography (micro-CT) imaging. To minimize the influence of water on imaging quality, samples were freeze-dried prior to scanning.

Imaging was conducted at an X-ray voltage of 60 kV and a current of 50 μA. A total of 1440 projection images were acquired with a detector resolution of 1920 × 1536 pixels, with each image exposed for 0.3 s. The voxel size was set at 0.62 μm, providing high spatial resolution to precisely visualize the internal pore structure and microarchitecture of the cryogel.

### 4.7. Polyacrylamide Gel Electrophoresis (SDS-PAGE)

Sodium dodecyl sulfate-polyacrylamide gel electrophoresis (SDS-PAGE) was performed using a 12% separating gel and a 5% stacking gel. Protein samples were mixed thoroughly with loading buffer and heated in a boiling water bath for 10 min to ensure complete denaturation. Denatured samples were precisely loaded into the wells using a micropipette.

Electrophoresis was initially carried out at a constant voltage of 80 V for the stacking gel to concentrate the proteins and form sharp bands. Subsequently, the voltage was increased to 120 V for the separating gel to achieve effective protein separation. Bromophenol blue was used as a tracking dye to monitor the progress of electrophoresis.

After electrophoresis, gels were stained with Coomassie Brilliant Blue R-250 on a shaker for 2 h. Excess stain was removed, and gels were destained until the background was clear and protein bands were distinctly visible. The stained and destained gels were photographed for further analysis and presentation.

### 4.8. Determination of Epoxy Group Density

A 1.0 g sample of freeze-dried cryogel was placed in a 25 mL conical flask. Subsequently, 10 mL of 1.3 mol/L sodium thiosulfate solution containing phenolphthalein as an indicator was added. The mixture was incubated at 37 °C for 30 min under continuous agitation. After incubation, the residual sodium thiosulfate was titrated with standardized 0.1 mol/L HCl solution. The endpoint was determined when the solution changed from red to colorless and remained stable for at least 30 s. The volume of HCl consumed was recorded for the calculation of epoxy group density [23].

(1) $$S=\frac{M_{HCl}\times \left(V_{0}-V_{1}\right)}{m}\times 100\%$$

where S is the epoxy density (mol/L), M_HCl_ is the concentration of HCl used (mol/L), V_0_ and V_1_ are the volumes of HCl before and after titration (mL), respectively, and m is the mass of the cryogel (g).

### 4.9. Cryogel Characterization

FT-IR Analysis: Freeze-dry the cryogel and grind to a fine powder. Mix thoroughly with spectroscopic-grade KBr, press into a transparent pellet, and record the infrared spectrum using an FT-IR spectrometer (Bruker Corporation, Billerica, MA, USA) [65].

SEM Imaging: Section the wet cryogel, freeze-dry to preserve microstructure, sputter-coat with metal, and observe surface morphology using a scanning electron microscope (JSM-6700F, JEOL Ltd., Tokyo, Japan). [44].

Swelling Performance: The cryogel was dried at 60 °C to a constant weight, and the dry mass was recorded as m_0_. The sample was then immersed in deionized water until equilibrium swelling was reached. After removing excess surface water by gentle blotting, the swollen mass was recorded as m_1_. Subsequently, the swollen cryogel was gently compressed to remove free water, and the remaining mass was recorded as m_2_. These parameters were used to evaluate the swelling degree and water-retention capacity of the cryogel [66].

The swelling degree of the cryogel was calculated using the following formula:

(2) $$Swelling\;Degree=\frac{m_{1}-m_{0}}{m_{0}}\times 100\%$$

The macroporosity of the cryogel was calculated using the following equation:

(3) $$Macroporosity=\frac{m_{1}-m_{2}}{m_{1}}\times 100\%$$

### 4.10. Other Assay Methods

Protein concentrations were determined using a Sigma BCA Protein Assay Kit (Sigma-Aldrich, St. Louis, MO, USA) according to the manufacturer’s instructions. Endotoxin levels in extracted lipopolysaccharide samples were quantified using a GenScript Endotoxin Detection Kit (Catalog No. L00350, GenScript Biotech Corporation, Nanjing, China).

### 4.11. Data Analysis

Unless otherwise specified, all experiments were performed in triplicate, and results are presented as mean ± standard deviation (Mean ± SD). Graphs were generated using Origin 8.0 and GraphPad Prism (version 10.4.0). Statistical analyses were conducted using one-way analysis of variance (ANOVA) followed by Tukey’s multiple comparison test. Differences were considered statistically significant at *p* < 0.05 and highly significant at *p* < 0.01.

## Figures and Tables

**Table 1:** Physicochemical properties of CG (HEMA-co-AM (CMCS)) (swelling ratio, porosity, epoxy group density after activation).

Cryogel Type	Swelling Ratio (%)	Porosity (%)	Epoxy Group Density (After Activation) (μmol/g)
Monolithic CG (HEMA-co-AM)	13.57	76.64	34.5
Stacked CG (HEMA-co-AM (CMCS))	13.96	75.83	41.17

**Table 2:** Basic information of various proteins (purification status, protein concentration, protein volume).

Protein Name	Purification Status	Protein Concentration	Protein Volume (mL)
SCP	YES	6.8	>75
AK	YES	4.96	>70
G20B (induced at 20 °C)	NO	15.39	>100
G20B (induced at 30 °C)	NO	10.1	>100
G20B (induced at 37 °C)	NO	8.32	>100
G37B (induced at 20 °C)	NO	18.00	>100
G37B (induced at 30 °C)	NO	10.86	>100
G37B (induced at 37 °C)	NO	9.77	>100

**Table 3:** Measurement of the effect of CG (HEMA-co-AM (CMCS))@ECH@PMB treatment on LPS adsorption in recombinant protein samples.

Protein Name	Before Treatment (EU/mL)	After Treatment (EU/mL)
SCP	6.3 × 104	0.2
AK	5.98 × 104	0.1
G20B (induced at 20 °C)	2.12 × 105	1.2
G20B (induced at 30 °C)	1.98 × 105	0.5
G20B (induced at 37 °C)	2.14 × 105	1.2
G37B (induced at 20 °C)	1.72 × 105	1.3
G37B (induced at 30 °C)	1.66 × 105	2.3
G37B (induced at 37 °C)	1.45 × 105	1.2

**Table 4:** Measurement of the effect of CG (HEMA-co-AM (CMCS))@ECH@PMB treatment on LPS adsorption in phage preparation samples.

Phage Name	Phage Type	Endotoxin Level Before Treatment (EU/mL)	Endotoxin Level After Treatment (EU/mL)
VPP1R	*Vibrio parahaemolyticus* phage	4.8 × 10^4^	242
HD1		3.2 × 10^4^	124
WM004		7.1 × 10^4^	23
MS1	*Salmonella* phage	1.5 × 10^5^	178
MS2		6.4 × 10^4^	87
MS3		8.5 × 10^4^	46
IME18	*Escherichia coli* phage	2.3 × 10^4^	228
MG16550		3.5 × 10^4^	32
VB SEqdws 315		7.1 × 10^4^	79

## Data Availability

The data presented in this study are openly available in this article/Supplementary Materials. The data presented in this study are available on request from the corresponding author.

## Supplementary Materials

The following supporting information can be downloaded at: https://www.mdpi.com/article/10.3390/gels12060470/s1, References [57,58,64] are cited in the Supplementary Materials.

## Abbreviations

The following abbreviations are used in this manuscript:

BSA	Bovine Serum Albumin
Hb	Hemoglobin
HSA	Human Serum Albumin
OVA	Ovalbumin
LYS	Lysozyme
