# Supplementary material for: Preparation of Stacked Polymyxin B-Functionalized Cryogels for Efficient Endotoxin Removal from Complex Biological Systems
Source: Gels. 2026 May 28;12(6):470. doi: 10.3390/gels12060470 (PMC13297916; doi:10.3390/gels12060470)
Supplement: Supplementary file 1 [file gels-12-00470-s001.zip › gels-4292685-supplementary.pdf]

Supplementary Materials:

S1. Preparation of Bacteriophage Preparations

S1.1. Amplification of Salmonella Phages (MS1, MS2, MS3)

To achieve efficient propagation of bacteriophages in specific host strains, 50 mL of LB liquid medium was used as the basal culture system. Briefly, 500 μL of pre-cultured host bacterial suspension was inoculated into the medium and thoroughly mixed by vortexing to ensure uniform distribution.

The culture was then incubated at 37 °C with shaking at 200 r/min for 2 h to allow moderate bacterial growth. Subsequently, 100 μL of phage suspension was added, followed by overnight incubation under the same conditions to enable phage amplification.After incubation, the culture was transferred into 50 mL centrifuge tubes and centrifuged at 8000 r/min for 15 min to collect the phage-containing supernatant. The supernatant was further filtered through a 0.22 μm sterile membrane to remove residual bacteria and impurities. The filtrate was finally collected and stored in sterile 50 mL serum bottles for subsequent use.

S1.2. Amplification of Escherichia coli Phages (IME18, MG16550, VB SEqdws315)

A total of 500 μL of pre-cultured host bacterial suspension was inoculated into 50 mL of LB liquid medium and mixed thoroughly by vortexing. The culture was incubated at 37 °C with shaking at 200 r/min for 2 h to promote host cell growth.

Subsequently, 100 μL of phage suspension was added, followed by overnight incubation under the same conditions for effective phage amplification. After cultivation, the culture was centrifuged at 8000 r/min for 15 min to separate the phage-containing supernatant.

The supernatant was then filtered through a 0.22 μm sterile membrane to remove residual cells and impurities. The filtered phage solution was collected and stored in sterile 50 mL serum bottles for further experiments.

S1.3. Amplification of Vibrio parahaemolyticus Phages (VPP1R, HD1, WM004)

A total of 500 μL of pre-cultured host bacterial suspension was inoculated into 50 mL of 2216E liquid medium and mixed thoroughly to ensure homogeneity. The culture was incubated at 37 °C with shaking at 200 r/min for 2 h to allow host cell growth.

Then, 100 μL of phage suspension was added, and the culture was incubated overnight under the same conditions to achieve phage propagation. After incubation, the culture was centrifuged at 8000 r/min for 15 min to collect the supernatant.

The supernatant was filtered through a 0.22 μm sterile membrane to remove residual host cells and impurities. The purified phage solution was collected and stored in sterile 50 mL serum bottles for subsequent use.

S1.4. Purification of Bacteriophage Preparations

The bacteriophage purification procedure was adapted from previously reported PEG precipitation and chloroform extraction methods with slight modifications [64]. Briefly, DNase I and RNase were added to the amplified phage suspension to final concentrations of 1 μL/mL each, followed by incubation at room temperature for 30 min to degrade free nucleic acids.Subsequently, NaCl was added to adjust the final concentration to 1 mol/L, and the suspension was incubated in an ice bath for 1 h to promote phage aggregation. The mixture was then centrifuged at 4 °C and 11,000 × g for 10 min, and the supernatant was collected.

Polyethylene glycol (PEG 6000) was added to the supernatant to a final concentration of 10%, followed by incubation in an ice bath for another 1 h. The suspension was centrifuged again under the same conditions, and the supernatant was discarded to obtain the phage pellet.The pellet was resuspended in an appropriate volume of physiological saline, followed by the addition of an equal volume of chloroform. After gentle mixing for 30 s, the mixture was centrifuged at 4 °C and 3000 × g for 10 min.Finally, the upper aqueous phase containing purified bacteriophages was collected and stored at 4 °C for further use.

S1.5. Determination of Phage Titer

The bacteriophage titer was determined using the standard double-layer agar plate method.Briefly, 100 μL of serially diluted phage suspension was mixed with an equal volume of host bacterial culture. The mixture was then added to a test tube containing 5 mL of semi-solid agar medium and gently mixed.Subsequently, the mixture was poured evenly onto a pre-prepared LB agar plate to form a double-layer structure. After incubation at 37 °C for 16–18 h, plaques were observed and counted, and the phage titer was calculated accordingly.

S2. Preparation of Recombinant Proteins

S2.1. Preparation of Enrofloxacin Antibody Proteins (G20B, G37B)

The expression of recombinant nanobody proteins was performed based on Liu with minor modifications [57]. E. coli carrying the recombinant pET-28a-SUMO nanobody expression vector was inoculated into LB liquid medium supplemented with kanamycin (30 μg/mL) and incubated at 37 °C in 1000 mL LB medium with shaking at 200 r/min for 10 h.

When the bacterial culture reached logarithmic phase (OD600 = 0.4–1.0), isopropyl β-D-1-thiogalactopyranoside (IPTG) was added to a final concentration of 5 μmol/L to induce expression, and the temperature was reduced to 28 °C for overnight induction.

After induction, cells were harvested by centrifugation at 4000 r/min and 4 °C for 15 min, and the pellet was resuspended in 0.01 M PBS buffer. The cells were lysed using high-pressure homogenization to release intracellular nanobody proteins. The lysate was centrifuged at 9000 r/min and 4 °C for 30 min to remove cell debris, and the

supernatant containing the target nanobody was collected. Protein concentration was determined for subsequent analysis.

S2.2. Preparation of Portunus trituberculatus Arginine Kinase (AK) and Sarcoplasmic Calcium-Binding Protein (SCP)

The expression of AK and SCP proteins was adapted from Zhu [58]. E. coli pET-28a-TM strains were streaked on LB agar plates and incubated at 37 °C for 12 h. Single colonies were picked and inoculated into 20 mL LB liquid medium containing kanamycin (50 μg/mL) and cultured at 37 °C for 10 h.

To obtain sufficient biomass, the starter culture was inoculated at a 1:1000 ratio into 1000 mL LB medium with kanamycin and incubated at 37 °C with shaking at 200 r/min for 4 h until OD600 reached approximately 0.6. IPTG was added to a final concentration of 1 μmol/L to induce protein expression, and cultures were incubated at 20 °C for 18–20 h.

Cells were harvested by centrifugation at 4000 r/min and 4 °C for 20 min, and the pellet was resuspended in 160 mL binding buffer. Cells were lysed using a high-pressure homogenizer. The lysate was centrifuged at 8000 r/min and 4 °C for 40 min to separate supernatant and pellet fractions. The pellet was resuspended in 1 mL binding buffer, and protein concentrations of both fractions were measured for further analysis.
